# When Gout Is in Doubt: A Curious Case of Lupus Nephritis

**DOI:** 10.7759/cureus.43737

**Published:** 2023-08-19

**Authors:** Sai Priya Maddury, Candice Reyes

**Affiliations:** 1 Internal Medicine, University of California, San Francisco (UCSF) Fresno, Fresno, USA; 2 Department of Rheumatology, California Department of Veterans Affairs, Fresno, USA

**Keywords:** gout, hmong, tophi, lupus nephritis, systemic lupus erythematosus

## Abstract

The association between systemic lupus erythematosus (SLE) and tophaceous gout is rarely documented. It can remain unnoticed if gout peculiarly lacks clinical symptoms. This may be attributed to treating SLE with immunosuppressive agents and steroids, which can mask the inflammation caused by gout. In this case report, we will discuss the case of a 35-year-old female from an indigenous Asian ethnic group called the Hmong community living in Fresno, California. She was diagnosed with lupus nephritis (LN) and was incidentally found to have tophi in the kidney with no gout symptoms clinically. This meant that persistent unchecked hyperuricemia could have been addressed if found earlier. This case study highlights potential genetic implications within the Hmong ethnicity in understanding gout when associated with SLE.

## Introduction

The chronic autoimmune illness “systemic lupus erythematosus" (SLE) can impact several organ systems. It exhibits various phenotypes, and its clinical manifestations range from modest mucocutaneous signs to severe multi-organ and central nervous system involvement [1]. It is marked by the accumulation of circulating antigen-antibody complexes in tissue, which triggers the release of inflammatory mediators and the inflow of inflammatory cells, culminating in clinically apparent kidney damage, most notably glomerulonephritis (GN). It leads to a serious complication of lupus nephritis (LN) in severe conditions, striking 40% of SLE patients [2]. Within 10 years of their LN, 20% of individuals proceed to end-stage renal disease (ESRD), albeit the survival rate has drastically improved after immunosuppressive therapy. As per the International Society of Nephrology/Renal Pathology Society (ISN/RPS), LN has been classified into six classes [3]. Class I (minimal mesangial LN) represents 20% of all the cases and exhibits normal glomerular appearance with no proteinuria. Class II (mesangial proliferative LN) makes up 7-22% of the cases and may depict mild proteinuria and hematuria, although the renal function might still be normal. In class III (focal LN), along with proteinuria and hematuria, the glomerular filtration rate (GFR) is also reduced. Class IV (diffuse LN) is the most prevalent and aggressive type, affecting more than 50% of glomeruli; patients with class IV LN are at the highest risk of progression to ESRD. Class IV LN requires intensive immunosuppression and usually includes high-dose steroids to reduce the activity of the immune system. Class V (membranous nephropathy) develops nephrotic-range proteinuria with elevated serum creatinine. Immune-complex deposits can be seen, majorly in the sub-epithelium, coupled with the loss of podocytes. In class VI (advanced sclerosing LN), more than 90% of the glomeruli turn sclerotic, significantly reducing renal function.

Gout is an inflammatory disease with joint pain, redness, and inflammation as the most common symptoms. Hyperuricemia is the basis for gout, and its persistence predisposes to recurrent gout flares and can lead to the formation of tophi (collections of uric acid). In some instances, these tophi are nephropathic in origin (especially medullary), which can lead to compromised renal function [4]. The treatment of gout involves uric acid-lowering medications, anti-inflammatory agents, lifestyle changes, and dietary modifications to manage symptoms and slow down disease progression. These measures can henceforth reduce the risk of further kidney damage and improve their quality of life.

The coexistence of LN and tophaceous gout in the kidney is infrequently noted [5-8]. Over 30 years, there have been only a handful of cases describing such a co-occurrence of LN and chronic gouty nephropathy. This co-occurrence can significantly impact a person’s health, as both diseases affect the kidneys, giving rise to compounding complications. Therefore, patients with both conditions must be diagnosed accurately, closely monitored for their symptoms, and given appropriate targeted treatment to avert potentially compounding complications, such as LN and chronic urate nephropathy with tophaceous renal deposits in these patients. Only with the recognition of its co-occurrence can healthcare providers ensure proper management and control of these conditions. In this article, we will discuss the case of a patient from the Hmong community suffering from SLE with LN who was incidentally found to have renal tophi on biopsy.

## Case presentation

A 35-year-old female patient of Hmong descent was diagnosed with SLE in 2009 when she presented with symptoms of progressive fatigue, hair loss for two months, four months of increasing discoid rashes, and a malar rash on the face. She also had occasional episodes of Raynaud’s phenomenon. In addition, she reported symptoms of joint pain and stiffness in the hands. On examination, she had active discoid lesions on the scalp (with scarring alopecia, especially on the frontal hairline) and around the nose, hands, knees, and toes. Her fingernails had become deformed, although she did not experience joint synovitis or tenderness. Over the next three years, she developed progressively worsening renal function. At that time, a renal biopsy showed mixed membranous diffuse proliferative lupus GN class IV with mild chronic tubulointerstitial changes. She was treated with an immunosuppression regimen of Plaquenil 200 mg twice daily and mycophenolate mofetil 500 mg twice daily. She was admitted to the hospital six years later for a lupus flare. 

Laboratory investigations showed pancytopenia with hemoglobin (Hb) 8.8 g/dl, white blood cell (WBC) 1.1 x 103 /µL, red blood cell (RBC) 3.01 x 106 /µL, platelets 99 x 103 /µL, and worsening renal function (serum creatinine 2.2 mg/dl, estimated glomerular filtration rate (eGFR): 27ml/min/1.73 m^2^) and proteinuria (600 mg protein in urine/day) along with a single episode of hematuria (blood in urine: +2). Serum lactate dehydrogenase (LDH) was elevated at 269 IU/L, direct Coombs test resulted in negative immunoglobulin G (IgG), antinuclear antibody (ANA) was positive at 8 IU/ml 1: 320 with a speckled and homogeneous pattern, Smith antibody IgG elevated at 133 AU/ml, U1 small nuclear ribonucleoprotein particle (snRNP) IgG elevated at 123 U, and Sjogren's syndrome (SS)-A IgG Abs were negative, as depicted in Table 1. Complement levels showed low C3 at 35.4 and normal C4 at 12.9. She had received one dose of intravenous (IV) cyclophosphamide (500 mg/m^2^ with prophylactic mesna) with pulse dose IV methylprednisolone. 

**Table 1:** Lab investigations WBC: white blood cells; RBC: red blood cells; eGFR: estimated glomerular filtration rate; LDH: lactate dehydrogenase; ANA: antinuclear antibody; U1 RNP/snRNP IgG: U1 small nuclear ribonucleoprotein particle (snRNP) IgG

Parameters	Detected value	Reference value
Hemoglobin	8.8 g/dl	12-16 g/dl
WBC	1.1 x 10^3^ /µL	4 -11 x 10^3^ /µL
RBC	3.01 x 10^6^ /µL	4-5.50 x 10^6^ /µL
Platelets	99 x 10^3^ /µL	140-440 x 10^3^ /µL
Serum creatinine	2.2 mg/dl	0.5- 1.1 mg/dl
eGFR	27ml/min/1.73 m^2^	90-120 ml/min/1.73m^2^
Urine protein	600 mg protein in urine/day	150 mg protein in urine/day
Serum LDH	269 IU/L	100-230 IU/L
ANA	8 IU/ml	>7.5 IU/ml
Smith antibody IgG	133 AU/ml	0-40 AU/ml
U1 RNP/snRNP IgG	123 U	0-40 U

**Figure 1:**
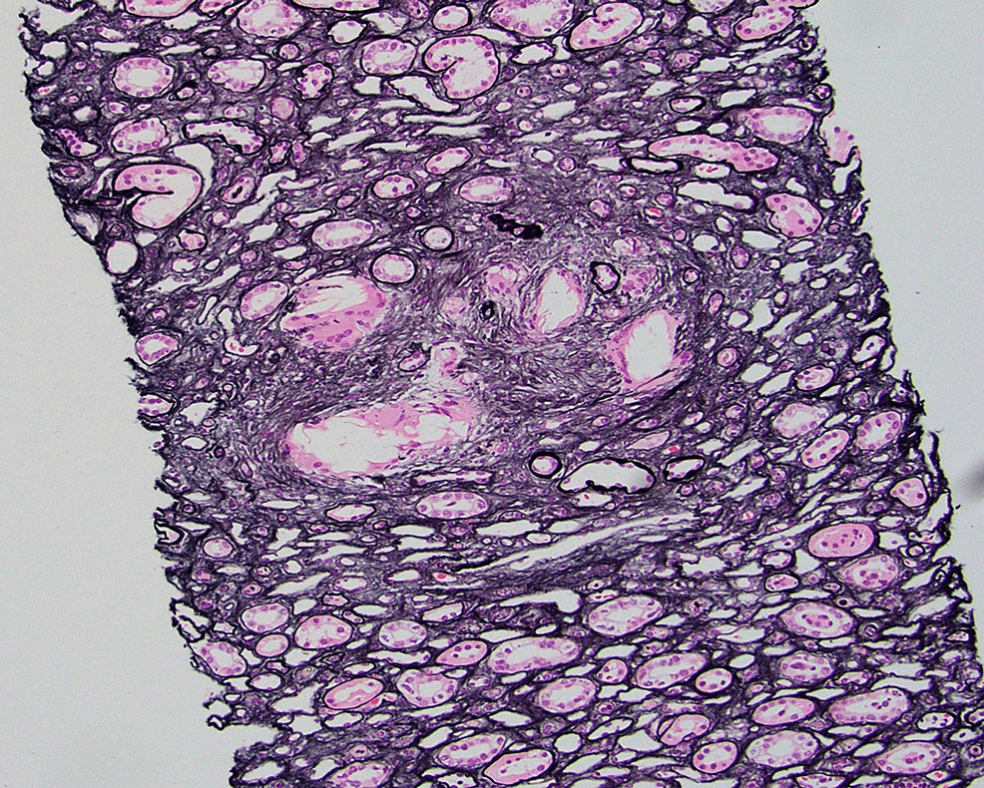
Light microscopy studied with hematoxylin and eosin (H&E), periodic acid-Shiff (PAS), Masson's trichome stain showing medullary gouty tophi consistent with chronic uric acid nephropathy

**Figure 2:**
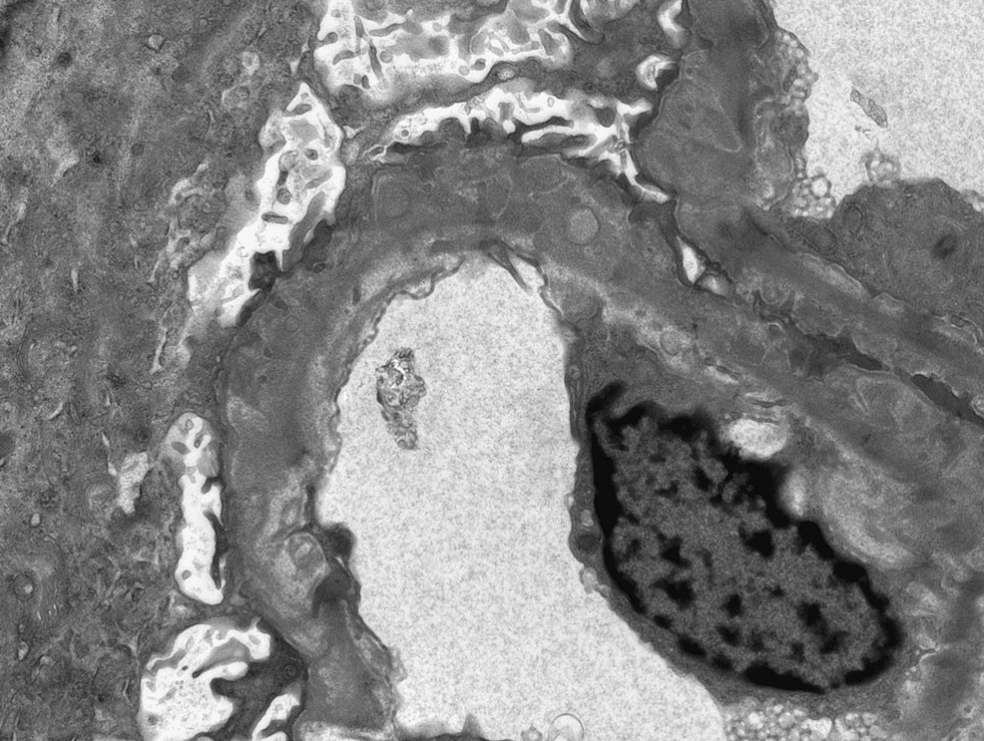
Electron microscopic image showing subepithelial deposits

A renal biopsy was performed, and on microscopic evaluation, five out of nine glomeruli were globally/near sclerotic. At least three glomeruli exhibited segmental sclerosis. All patient loops exhibited conspicuous spike/pinhole formation; many capillary loops demonstrated double contours. Mesangial regions were widened by a matrix and deposits with mild segmental cellularity. The injury pattern was membranous nephropathy (class V lupus) and chronic diffuse sclerosing LN (class IV lupus). In addition, there were focal features of mild endocapillary hypercellularity and rare large subendothelial immune complex deposits. Features of chronicity were seen, including diffuse global glomerulosclerosis, focal segmental glomerulosclerosis, and mild cortical scarring. Incidentally, medullary gout tophi consistent with chronic uric acid nephropathy were noted (Figures 1, 2).

The patient never had any history of mono- or oligo-articular joint pain, redness, or joint swelling. Her diet consisted primarily of white meat with occasional consumption of red meat. She never consumed alcohol. The patient denied any family history of gout. Her labs showed an elevated uric acid of 14 mg/dl (N: 2.6-6.0 mg/dl) after her renal function declined. However, her baseline uric acid levels were not checked before this. 

Since her admission to the hospital for acute lupus flare, the patient has been on a prednisone tapered dose of 60 mg/day, which was eventually tapered over months, and she has then been maintained on hydroxychloroquine 600 mg twice a day and mycophenolate mofetil 500 mg twice a day.

She was started on daily peritoneal dialysis for her ESRD in 2019. Her latest labs are hemoglobin (Hb) 7.7 g/dl, WBC 12.3 x 103 /µL, RBC 3.37 x 106 /µL, platelet 225 x 103 /µL, Na 139 mEq/L, K 2.8 mEq/L, and creatinine 9.6 mg/dl. Currently, she is awaiting renal transplantation.

## Discussion

This patient meets the criteria for the diagnosis of SLE based on the Systemic Lupus International Collaborating Clinics Criteria. She had alopecia, discoid lupus on the scalp confirmed by biopsy, anemia, thrombocytopenia, serological evidence with positive ANA, dsDNA, and biopsy-confirmed LN class IV. She had no clinical symptoms of gout, including monoarticular pain or tophi in joints. However, she was noted to have elevated serum uric acid when she presented with renal disease. The kidney biopsy revealed medullary gouty tophi consistent with chronic uric acid nephropathy. A prior biopsy six years prior did not show any evidence of tophi. The fact that this patient had a significant enough burden of uric acid to deposit tophi in the kidney and to cause chronic uric acid nephropathy without any systemic symptoms of gout is very interesting but also concerning to be missed and leave untreated. 

Based on the medical literature review, a patient's concurrence of SLE and gout is quite rare [6-8]. The rare occurrence of such a coexistence could be due to a wide disparity in age and gender demographics. Gout occurs most often in men (20:1) and older people (ages 30 to 50 years), whereas SLE usually affects younger females (ages 15 to 27 years) [9]. Lalley et al. postulated that SLE might confer protection against gout. Although monosodium urate is an activator of the complement system, these authors speculated that patients with hypocomplementemia, such as those with SLE, might have a diminished inflammatory response to monosodium urate [10].

A similar case was reported by Navarra et al., in which a patient had biopsy-proven LN with renal micro tophi [11]. However, in that case, the patient had gouty arthritis symptoms preceding the diagnosis of lupus. Hence, this case is unique, where medullary gout tophi consistent with chronic uric acid nephropathy was noted on biopsy co-existing with LN without any history of gout arthropathy. It is postulated that immunosuppressive therapy can inhibit or dampen the clinical expression of gout even while urate deposits accumulate in the joint or soft tissues, which could be why the patient never had any gout-related symptoms. Moreover, women frequently present with insidious polyarthritis rather than acute monoarthritis [12-14]. 

Tophi generally develop in chronic gout after many years of typical acute episodic arthritis [15]. The development of tophi before gouty arthritis is unusual. Wernick et al. reported six patients who developed tophi before or without clinical gout at the Veterans Affairs Medical Center (Portland, Ore). Based on their literature review, only 26 well-documented cases were noted [16]. This patient not only had medullary tophi but also had a severe burden to cause chronic urate nephropathy. This patient belongs to the Hmong community in Fresno, California. The Hmong are an Asian ethnic group originally from southern China that later migrated to the northern regions of Laos, Vietnam, and Thailand. They have a higher prevalence of gout and gout-related comorbidities than non-Hmong communities. Several studies have been conducted to investigate gout and hyperuricemia among the Hmong. For example, it was found that the Hmong have a twofold higher prevalence of gout compared to the non-Hmong population in Minnesota [17]. In addition, specific alleles associated with an increased risk of hyperuricemia are found more frequently in Hmong individuals than in individuals of European or Han Chinese descent. The specific polymorphisms found in the Hmong people also provide a basis for anecdotal reports, suggesting that allopurinol may not be effective when used in Hmong patients, as these alleles code for uric acid transporters in the proximal convoluted tubule. Therefore, uric acid levels may not decrease with xanthine oxidase inhibitors [18]. This observation can likely represent the genetic basis for the Hmong’s higher prevalence of hyperuricemia and gout.

## Conclusions

This is a rare case of finding incidental tophaceous deposits in the kidney in a patient with LN without gout symptoms. However, the patient’s ethnicity (Hmong cultural background) is likely an independent risk factor for gout, which has been observed clinically in a few studies. This finding warrants further studies on this community’s genetic factors associated with severe gout and clarifies whether the Hmong population is at increased risk of concurrently suffering from SLE and gout.
